# Teneurin-3 regulates the generation of non-image-forming visual circuitry and responsiveness to light in the suprachiasmatic nucleus

**DOI:** 10.1371/journal.pbio.3002412

**Published:** 2023-12-04

**Authors:** John L. Hunyara, K. M. Daly, Katherine Torres, Maria E. Yurgel, Ruchi Komal, Samer Hattar, Alex L. Kolodkin

**Affiliations:** 1 The Solomon H. Snyder Department of Neuroscience, Kavli Neuroscience Discovery Institute, The Johns Hopkins University School of Medicine, Baltimore, Maryland, United States of America; 2 Section on Light and Circadian Rhythms (SLCR), National Institute of Mental Health, National Institutes of Health, Bethesda, Maryland, United States of America; 3 Department of Biology, Johns Hopkins University, Baltimore, Maryland, United States of America; Yale University, UNITED STATES

## Abstract

Visual system function depends upon the elaboration of precise connections between retinal ganglion cell (RGC) axons and their central targets in the brain. Though some progress has been made in defining the molecules that regulate RGC connectivity required for the assembly and function of image-forming circuitry, surprisingly little is known about factors required for intrinsically photosensitive RGCs (ipRGCs) to target a principal component of the non-image-forming circuitry: the suprachiasmatic nucleus (SCN). Furthermore, the molecules required for forming circuits critical for circadian behaviors within the SCN are not known. We observe here that the adhesion molecule teneurin-3 (Tenm3) is highly expressed in vasoactive intestinal peptide (VIP) neurons located in the core region of the SCN. Since Tenm3 is required for other aspects of mammalian visual system development, we investigate roles for Tenm3 in regulating ipRGC-SCN connectivity and function. Our results show that Tenm3 negatively regulates association between VIP and arginine vasopressin (AVP) neurons within the SCN and is essential for M1 ipRGC axon innervation to the SCN. Specifically, in *Tenm3*^*-/-*^ mice, we find a reduction in ventro-medial innervation to the SCN. Despite this reduction, *Tenm3*^*-/-*^ mice have higher sensitivity to light and faster re-entrainment to phase advances, probably due to the increased association between VIP and AVP neurons. These data show that Tenm3 plays key roles in elaborating non-image-forming visual system circuitry and that it influences murine responses to phase-advancing light stimuli.

## Abstract 2

What enables intrinsically photosensitive retinal ganglion cells to target the suprachiasmatic nucleus? This study demonstrates that the adhesion molecule teneurin-3 plays a key role in elaborating this circuitry and influences murine responses to phase-advancing light stimuli.

## Introduction

The initial processing of visual information occurs in the retina, which is a light sensitive central nervous system (CNS) tissue located at the back of the eye. Photons of light activate photoreceptors, which then pass visual information to retinal ganglion cells (RGCs) via bipolar cells. RGC axons then propagate visual information via the optic nerve to the brain, where higher order processing occurs to produce meaningful visual representations or behavior. Therefore, to ensure that the retina conveys visual perception and tracking, and luminance coding throughout life, it is critical that precise connections between >40 distinct RGC subtypes [1] and over 50 central brain targets [2] are formed during development.

RGC-central target connectivity is established in a stereotyped manner, with neural guidance cues and cell adhesion molecules regulating RGC axon innervation of retinorecipient centers. For example, cadherin-6 is required for innervation of retino-recipient targets such as the olivary pretectal nucleus (OPN) and the medial division of the posterior pretectal nucleus [3]. Further, semaphorin 6A is expressed in subsets of direction-selective RGCs and is required for recognizing plexin receptor ectodomains located in the medial terminal nucleus and for establishing connections critical for accessory optic system-mediated image stabilization on the retina [4]. Additionally, reelin, a secreted extracellular matrix protein, is required in the intergeniculate leaflet (IGL) and ventral lateral geniculate nucleus for normal targeting by intrinsically photosensitive retinal ganglion cells (ipRGCs) [5].

Though progress has been made in identifying the molecules required in some retino-recipient targets for RGC connectivity, very little is known about innervation of most central targets, including the suprachiasmatic nucleus (SCN). The SCN is in the ventral hypothalamus and is entrained to light via ipRGCs to regulate circadian rhythms throughout the body [6]. It is comprised of multiple, spatially segregated, GABAergic neuron subtypes that are largely defined by differences in neuropeptide expression [7]. The principal SCN neuron types are arginine vasopressin (AVP)-secreting neurons located in the shell, as well as gastrin releasing-peptide (GRP)-secreting and vasoactive intestinal peptide (VIP)-secreting neurons located in the core [8]. A recent study shows that in mutants lacking the receptors for AVP, faster entrainment to a jet-lag paradigm is observed [9], suggesting that AVP acts as a brake for fast changes in the phase of the clock in response to light.

It was long thought that VIP neurons were the principal cell type to receive direct retinal input [10]; however, recent evidence shows that AVP and GRP cells are also synaptically connected to ipRGCs [11]. Therefore, it is possible that a diverse set of molecules is required to properly establish ipRGC synaptic connectivity onto a heterogeneous population of retino-recipient cells that are critical for non-image-forming visual functions, such as circadian photoentrainment and pupil constriction.

Here, we consider the role of a teneurin family member, teneurin-3 (Tenm3), in regulating SCN innervation by ipRGCs, intra-SCN connectivity, and aspects of circadian photoentrainment. Teneurins are a family of single-pass, type II transmembrane proteins that are important for many aspects of neuronal wiring across species. In *Drosophila*, trans-synaptic homophilic interactions between teneurins establish wiring specificity in both the olfactory system [12] and at the neuromuscular junction [13]. Of the 4 mammalian teneurins (teneurins 1–4), Tenm3 homophilic interactions are critical for hippocampal CA1 neuron connectivity to the distal subiculum [14]. These precise limbic system connections are further regulated by Tenm3 repulsive interactions with latrophilin-2, which is expressed in the proximal subiculum [15]. These observations demonstrate the importance of teneurin homophilic and heterophilic interactions during neural circuit development.

Various roles for teneurins in mediating neuronal connectivity during vertebrate visual system development have been appreciated for some time [16]. *Tenm3* knockdown in the developing zebrafish visual system leads to neurite lamination errors in both the inner plexiform layer and the tectum [17]. In mammals, Tenm3 homophilic interactions underlie accurate topographic mapping of retinal projections in both the lateral geniculate nucleus and superior colliculus (SC) [18,19]. As a result of these impairments, *Tenm3*^*-/-*^ mice perform poorly in a visual cliff test that assesses an animal’s depth perception [18]. Additionally, *Tenm2*^*-/-*^ mice have severely reduced ipsilateral input to both the dorsal lateral geniculate nucleus and SC [20], suggesting that in addition to Tenm3, Tenm2 is required for proper development of binocular vision. However, whether teneurins play similar roles in establishing neuronal connectivity in non-image-forming visual targets, like the SCN, is unknown.

## Results

To identify novel candidate molecules with the potential to regulate ipRGC-SCN connectivity, we conducted an *in silico* screen using the Allen Brain Atlas, a publicly available resource that includes *in situ* hybridization data at several stages of mouse brain development [21]. We began by searching for genes with high expression in the developing SCN that encode neural guidance or cell adhesion molecules. From this analysis, we observed robust expression of the teneurin family member *Tenm3* in the postnatal SCN that persisted into adulthood. Given the requirement for Tenm3 in other aspects of mammalian visual system development and function [18,19], we chose to characterize *Tenm3* expression in the SCN and to investigate roles for Tenm3 in regulating ipRGC-SCN connectivity and function.

**Fig 1:**
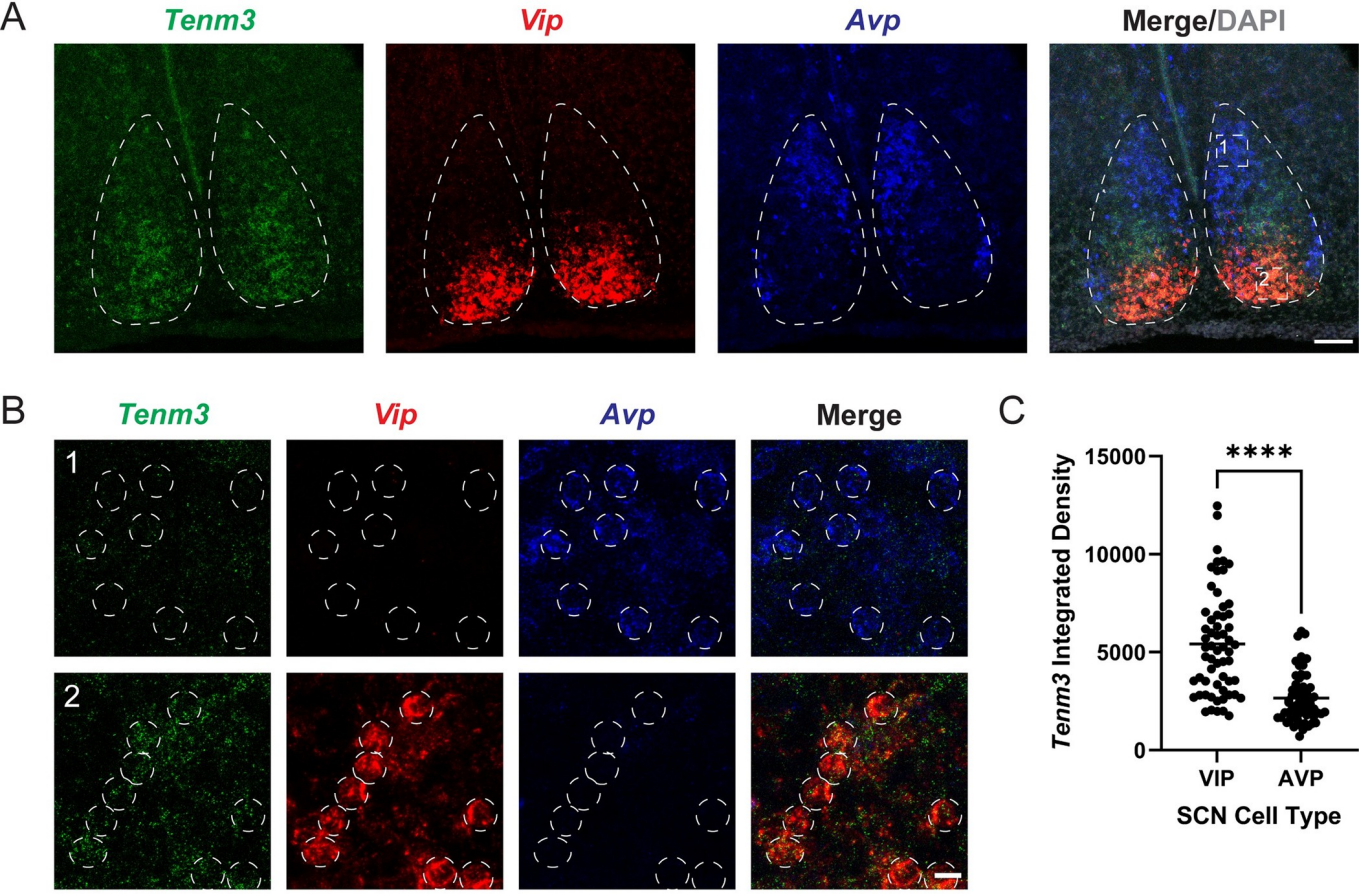
*Tenm3* expression in the SCN is highest in VIP cells. (A) RNAscope in situ hybridization in coronal sections of P5 *C57Bl/6J* brains for *Tenm3*, *Vip*, and *Avp*. Dotted white line demarcates the SCN border. *n* = 3 mice per group. Scale bar = 100 μm. (B) Magnified images corresponding to boxes 1 and 2 in Merge/DAPIA. Note weak *Tenm3* expression in AVP neurons (indicated by dotted white circles in box 1), but much stronger expression in most VIP neurons (indicated by dotted white circles in box 2). Scale bar = 25 μm. (C) *Tenm3* expression is higher in VIP neurons compared to AVP neurons. Lines represent the mean. Symbols represent individual cells (see S1 Data). *n* = 3 mice and 20 cells/mouse. Statistics: unpaired *t* test. ****p < 0.0001. AVP, arginine vasopressin; SCN, suprachiasmatic nucleus; VIP, vasoactive intestinal peptide.

Our *in silico* observations suggested that *Tenm3* expression in the developing SCN is highest in the core region. We confirmed this by performing RNAscope *in situ* hybridization [22] on P5 *C57Bl/6J* brain sections with probes designed to detect *Tenm3*, *Vip*, and *Avp*, observing that *Tenm3* expression is strongest in the SCN core region and is highly expressed in VIP cells (Fig 1A–1C and S1 Data). AVP cells that occupy the shell of the SCN also expressed *Tenm3*, but at much lower levels (Fig 1A–1C and S1 Data). Additionally, we found that a subset of non-AVP, non-VIP cells in the central SCN express *Tenm3*. We speculate that these cells are likely GRP cells, which are known to reside in this region. Taken together, we show that although multiple cell types in the postnatal SCN express *Tenm3*, it is expressed at much higher levels in VIP neurons.

Since Tenm3 homophilic interactions are important for axon targeting and topographic mapping in other regions of the developing visual system [18,19], we investigated this possibility for the ipRGC-SCN neural circuit. Using RNAscope *in situ* hybridization, we found that *Tenm3* is very weakly expressed in the SCN-innervating *Pou4f2-*negative ipRGCs and is modestly expressed in *Pou4f2*-positive ipRGCs (S1A Fig and S1 Data). We confirmed our in situ expression data by performing transcriptomic profiling using single-cell RNA sequencing (scRNA-seq) on GFP-positive cells from *Opn4*^*Cre/+*^*; Brn3b*^*zDta/+*^*; Rosa26*^*fsTRAP/+*^ retinas isolated by FACS at 2 early postnatal time points. Our scRNA-seq data reveals minimal *Tenm3* expression in the *Pou4f2*-negative cluster of sequenced P1 and P5 ipRGCs (S1B–S1E Fig). In contrast, we found that expression of the latrophilins (*Adgrl1*, *Adgrl2*, and *Adgrl3*) is variable across ipRGC types. In the *Pou4f2*-negative cluster of sequenced P1 and P5 ipRGCs, almost all cells express *Adgrl1*, whereas expression of *Adgrl2* and *Adgrl3* is more heterogenous. These findings suggest that any role for Tenm3 in establishing ipRGC-SCN connectivity is likely to occur through heterophilic interactions with latrophilins, or with an as yet unidentified binding partner, rather than through homophilic Tenm3 associations.

**Fig 2:**
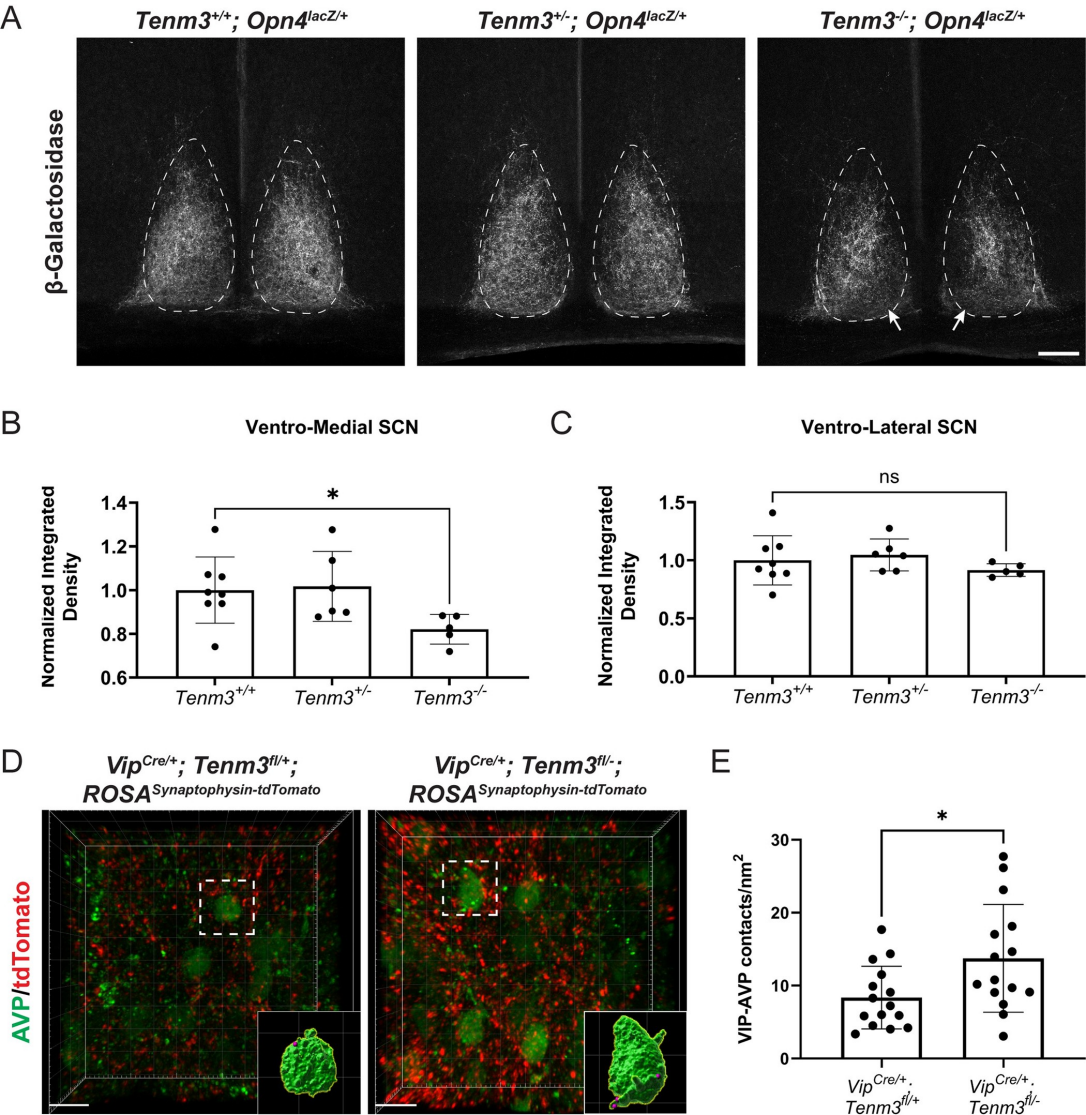
ipRGC axon SCN innervation and intra-SCN synaptic connectivity are altered in *Tenm3*^*-/-*^ mice. (A) β-Galactosidase labeling is weaker in the ventro-medial region of the SCN (arrows) in *Tenm3*^*-/-*^*; Opn4*^*lacZ/+*^ mice compared to *Tenm3*^*+/-*^*; Opn4*^*lacZ/+*^ mice at P40. Scale bar = 100 μm. (B, C) The ventro-medial, but not the ventro-lateral, SCN is hypo-innervated in *Tenm3*^*-/-*^*; Opn4*^*lacZ/+*^ mice. Lines represent mean and SD. Symbols represent individual mice (see S1 Data). Statistics: unpaired *t* test. *p < 0.05. (D) Maximum intensity projection of the SCN in control and *Tenm3* conditional mutants showing distribution of VIP-positive presynaptic terminals in relation to AVP cells. Dotted white boxes denote rendered cells with synaptic contacts shown in insets. Scale bars = 15 μm. (E) The density of VIP-AVP synaptic contacts in the SCN is greater in *Tenm3* conditional mutants as compared to controls. Lines represent mean and SD. Symbols represent individual cells (see S1 Data). *n* = 4 mice per genotype. Statistics: unpaired *t* test. *p < 0.05. AVP, arginine vasopressin; ipRGC, intrinsically photosensitive retinal ganglion cell; SCN, suprachiasmatic nucleus; VIP, vasoactive intestinal peptide.

Our observation that Tenm3 is highly expressed in the developing SCN led us to ask whether Tenm3 is required for M1 ipRGC innervation of the SCN. Therefore, we acquired *Tenm3*^+/-^ mice [18], generated *Tenm3*^*-/-*^*; Opn4*^*lacZ/+*^ mice, and quantified M1 ipRGC axon innervation of the SCN. We found that the ventro-medial (core) region of the SCN in *Tenm3*^*-/-*^*; Opn4*^*lacZ/+*^ mice shows reduced M1 ipRGC innervation (Fig 2A and 2B and S1 Data), whereas innervation of the ventro-lateral SCN is normal (Fig 2C and S1 Data). This suggests that Tenm3 is only required in specific cell populations of the SCN, most likely VIP neurons, for proper axon targeting. Importantly, we found that M1 ipRGC innervation of other central targets, such as the IGL and OPN shell, is normal (S2 Fig and S1 Data). As suggested by our expression data, conditional removal of Tenm3 from ipRGCs does not affect ipRGC innervation of the SCN (S3A–S3C Fig and S1 Data), supporting a role for SCN-derived Tenm3 in regulating ipRGC innervation. Taken together, Tenm3 is not required for central target innervation by all M1 ipRGCs but is essential for M1 ipRGC axon innervation of select cell types within the SCN.

One explanation for these innervation deficits is that in the absence of Tenm3, conventional RGCs lose their responsiveness to a repulsive factor and instead target the SCN, replacing ipRGCs. To test this, we injected a retrograde virus into the SCN of *Tenm3*^*-/-*^ mice and performed immunohistochemistry on retrogradely labeled retinas to identify ipRGCs. We quantified the percentage of retrogradely labeled melanopsin-positive and melanopsin-negative RGCs and found no difference between *Tenm3*^*+/-*^ and *Tenm3*^*-/-*^ mice (S4 Fig and S1 Data).

Since there is known synaptic connectivity among SCN subtypes, and since our observations suggest heterophilic interactions involving Tenm3 are required for normal ipRGC innervation of the SCN, we wondered whether synaptic connectivity between cells within the SCN (intra-SCN connectivity) is also perturbed in the absence of Tenm3. Since VIP cells express the highest levels of *Tenm3* in the SCN, we assessed the consequences of conditional *Tenm3* deletion from VIP cells on intra-SCN connectivity. We generated *VIP*^*Cre/+*^*; Tenm3*^*fl/-*^*; ROSA*^*Synaptophysin-tdTomato/+*^ mice to label sites of very close contact between VIP cells and other cell types within the SCN, an association that likely reflects synaptic connectivity (Fig 2D). We quantified VIP-AVP close contacts and observed an increase in their number in *VIP*^*Cre/+*^*; Tenm3*^*fl/-*^*; ROSA*^*Synaptophysin-tdTomato/+*^ mice compared to controls (Fig 2E and S1 Data). This result suggests that Tenm3 also plays a critical role in regulating connectivity within the SCN.

**Fig 3:**
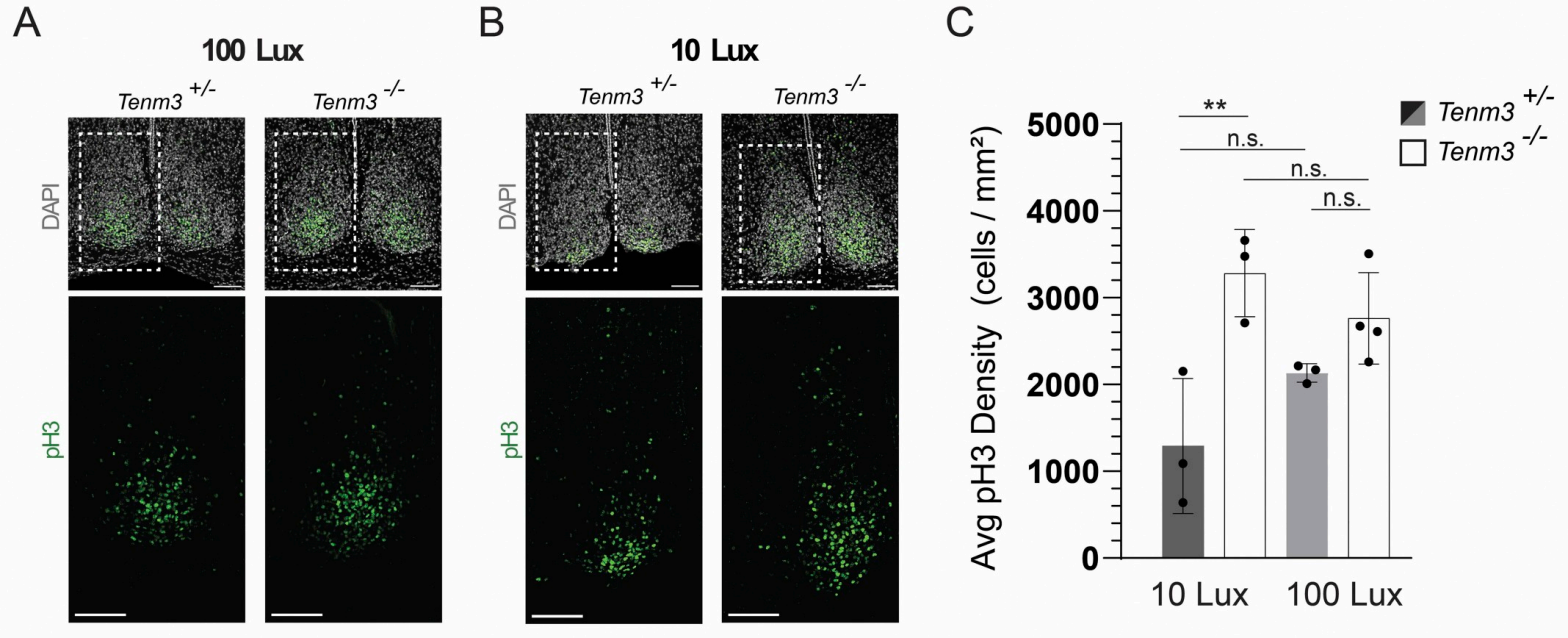
Light-induced H3 phosphorylation is enhanced in the SCN of *Tenm3*^*-/-*^ mice. (A) Representative images depicting phosphorylation of H3 in response to a 100 lux light pulse in *Tenm3*^*+/-*^ and *Tenm3*^*-/-*^ mice. Composite maximum projection images of bilateral SCN sections depict DAPI-stained nuclei pseudo-colored gray and pH3-positive cells in green. Regions in white dotted-line boxes depict pH3 immunoreactivity in the single SCN hemisphere. Scale bars = 100 μm. (B) Representative images of pH3 immunoreactivity in response to a 10 lux light pulse. (C) Quantification of pH3-positive cell density shows a significant increase in *Tenm3*^*-/-*^ mice following a 10 lux light pulse. *p* = 0.0061, *n* = 3–4 mice per group, error shown as standard deviation (see S1 Data). One-way ANOVA with Tukey’s correction for multiple comparisons. SCN, suprachiasmatic nucleus.

Our observations of altered ipRGC-SCN innervation and intra-SCN connectivity in *Tenm3*^*-/-*^ mice prompted us to ask whether there are functional differences in how light is processed within the SCN between wild-type and *Tenm3*^*-/-*^ mice. First, we investigated whether light-induced cellular activation is affected by the loss of Tenm3. We entrained *Tenm3*^*+/-*^ and *Tenm3*^*-/-*^ mice to a 12-h light-dark cycle. Once entrained, mice were released into darkness for 1 day and then presented with a 15-min light pulse at circadian time (CT) 22. We used the phosphorylation of histone H3 (pH3) as a readout since recent work shows that this rapid phosphorylation event in the SCN does not exhibit a circadian component and is only activated by light [23,24]. At first, we found no difference in pH3 immunoreactivity between *Tenm3*^*+/-*^ *and Tenm3*^*-/-*^ mice when they were presented with a light pulse of 100 lux (Fig 3A and 3C and S1 Data). However, we reasoned that this intensity could be saturating, and so we next presented mice with a light pulse of 10 lux and observed significantly increased pH3 immunoreactivity in the core and the shell of the SCN in *Tenm3*^*-/-*^ mice (Figs 3B, 3C, and S5 and S1 Data). Interestingly, pH3-positive cell density in *Tenm3*^*-/-*^ mice was elevated under both dim and saturating light intensities (Fig 3B and 3C and S1 Data), suggesting that the SCN has increased sensitivity to external light cues in the absence of Tenm3.

**Fig 4:**
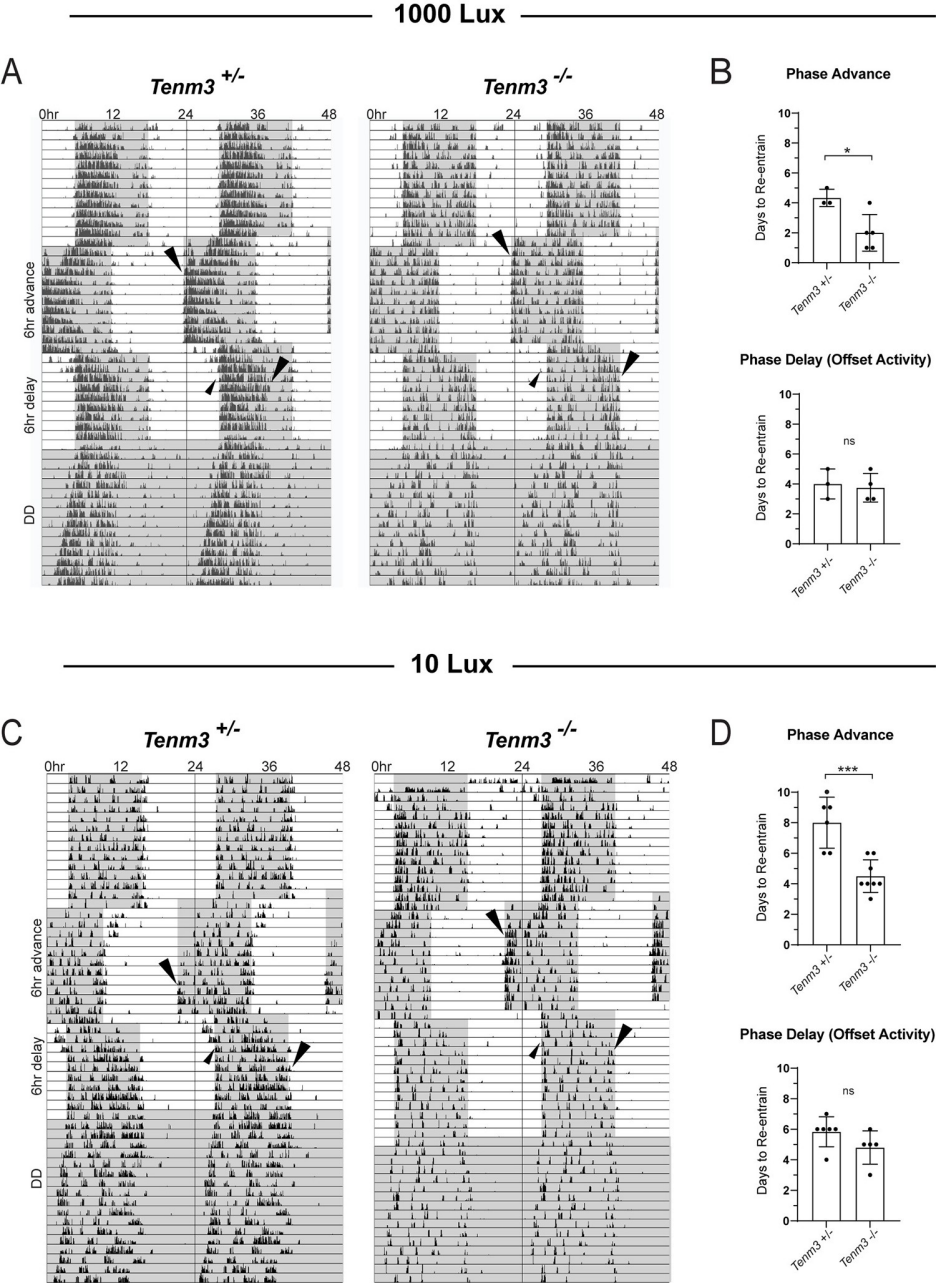
Circadian phase-advances are accelerated in *Tenm3*^*-/-*^ mice at 1,000 lux and 10 lux. (A) Representative double-plotted actograms show wheel-running activity throughout a 6-h phase advance and subsequent delay under 12:12 LD conditions at 1,000 lux. (B) At 1,000 lux, *Tenm3*^*+/-*^ mice require 4 to 5 days to stably re-entrain to a phase advance. *Tenm3*^*-/-*^ mice re-entrain to phase advances at an accelerated rate (*p* = 0.0115), but not phase delays. (C, D) Significantly accelerated re-entrainment to phase advances (*p* = 0.0002) is even more pronounced at 10 lux light intensities while re-entrainment to phase delays remains normal. Arrows denote observed day of re-entrainment to advances and delays (measured by onset and offset of activity). Error represents standard deviation (see S2 and S3 Data). One-tailed unpaired *t* test. LD, light-dark.

Increased SCN cell activation in response to light stimuli in *Tenm3*^*-/-*^ mice led us to hypothesize that the loss of Tenm3 may also affect how the SCN adjusts circadian rhythms in response to changes in the light environment. Therefore, we employed an artificial jet-lag wheel-running assay to assess whether *Tenm3*^*-/-*^ mice can properly re-entrain their endogenous circadian rhythms to the external light environment compared to heterozygous controls. *Tenm3*^*-/-*^ mice showed normal circadian photoentrainment (Fig 4A and S2 Data). However, when the light cycle was advanced by 6 h, *Tenm3*^*-/-*^ mice re-entrained to the new light pattern within an average of 2 days, whereas it took *Tenm3*^*+/-*^ mice approximately 4 days to re-entrain, similar to the normal re-entrainment speed of published wild-type animals [25,26] (Figs 4A, 4B, S6A, S6C, and S6E and S2 Data). When the light cycle was delayed by 6 h, we observed no differences in the time required to re-entrain between *Tenm3*^*+/-*^ and *Tenm3*^*-/-*^ mice (Figs 4A, 4B, S6F, and S6G and S2 Data). Furthermore, *Tenm3*^*-/-*^ animals exhibited a normal circadian period under constant dark conditions, indicating that the circadian clock is not disrupted in these mutants (S6H Fig and S2 Data).

We next reasoned that if loss of Tenm3 results in increased sensitivity to external light cues, then *Tenm3*^*-/-*^ mice would still be able to re-entrain to low light phase advances at an accelerated rate. To test this, we performed a wheel-running jet-lag assay under 10 lux, 2 orders of magnitude dimmer than the previous light cycle we tested. We observed that all mice were still able to entrain to the 10 lux light-dark cycle (Figs 4C and S7 and S3 Data). Additionally, we found that *Tenm3*^*-/-*^ mice re-entrain to phase advances significantly faster, requiring an average of 4.5 days compared to *Tenm3*^*+/-*^ mice, which took an average of 8 days to re-entrain to a 6-h phase advance under 10 lux (Fig 4D and S3 Data). Consistent with previous results, we observed no significant difference in re-entrainment to delays (Fig 4D and S3 Data).

Finally, we asked whether the responses to phase shifting in *Tenm3*^*-/-*^ mice are due to melanopsin-mediated phototransduction. We generated *Tenm3*^*-/-*^*; Opn4*^*Lacz/Lacz*^ double knockout (DKO) mice and found that the combined loss of melanopsin and Tenm3 in the DKO mice does not result in slower re-entrainment to an advanced light pulse (S6D Fig and S2 Data), despite findings in *Opn4*^*-/-*^ mice [27,28] showing a trend toward slower re-entrainment to both advances and delays (S6B, S6E–S6G Fig and S2 Data). These data suggest that melanopsin is not required for the Tenm3-based regulation of SCN responses to phase advances.

## Discussion

Data presented in this study suggest that the cellular adhesion molecule Tenm3 plays an important role in SCN connectivity without disrupting the circadian clock and, specifically, influences the response to phase-advancing light stimuli. These results are the first to show that Tenm3 is critical for establishing M1 ipRGC axon innervation to select SCN cell types and for regulating VIP-AVP connectivity within the SCN. Loss of Tenm3 has profound effects on the sensitivity of the SCN since *Tenm3*^*-/-*^ mice exhibit increased apparent VIP-AVP synaptic connectivity and increased sensitivity to dim light stimuli. Thus, a unique behavioral response of *Tenm3*^*-/-*^ mice is resistance to “jet-lag” induced by phase-advancing, but not phase-delaying, light cues.

If Tenm3 is required for SCN development along with maintenance and/or function in the adult, why do *Tenm3*^*-/-*^ mice rapidly re-entrain to phase advances? Interestingly, removal of the AVP receptors V1a and V1b leads to rapid re-entrainment similar to what we observe in *Tenm3*^*-/-*^ mice [9]. In contrast to our observations in *Tenm3*^*-/-*^ mice, however, *V1a*^*-/-*^*; V1b*^*-/-*^ mice rapidly re-entrain to both phase advances and delays. This can be mimicked through pharmacological blockade using V1a and V1b antagonists, suggesting that AVP-mediated inter-neuronal communication underlies the timing of re-entrainment observed in wild-type animals to a jet-lag paradigm [9]. If *Tenm3* is only weakly expressed in AVP neurons, how then could it play a significant role in AVP signaling? Recent studies identify a high degree of synaptic connectivity not only among similar SCN cell types, but also between distinct neuronal populations in the SCN core and shell [29]. Notably, almost all VIP neurons form multiple synaptic contacts onto AVP cells [29]. Therefore, one hypothesis is that loss of Tenm3 in VIP cells alters VIP-AVP connectivity, consistent with observations in this study, thus influencing AVP signaling and leading to rapid re-entrainment following phase shifts.

AVP signaling is equally important for re-entrainment following both phase advances and delays [9]. Our unexpected observation that *Tenm3*^*-/-*^ mice rapidly re-entrain to phase advances, but not delays, raises the following question: How can the distinct behavioral responses to phase shifts we observe in *Tenm3*^*-/-*^ mice be explained at the molecular level? One possibility that is gaining recent support in the field is that there are multiple signals and/or circuits which mediate the phase advancing versus delaying effects of light on the circadian clock [30,31]. Consistent with this idea, only phase-advancing, but not delaying, light stimuli activate the SCN shell, where the AVP neurons reside [23]. This suggests that AVP cells found in the SCN shell are preferentially activated only in response to phase-advancing light and is consistent with activating more AVP neurons having the opposite effect as observed in *V1a*^*-/-*^*; V1b*^*-/-*^ mice. Therefore, our prediction is that the increased synaptic inhibitory input from VIP onto AVP neurons we observe in *Tenm3*^*-/-*^ mice should lower AVP activation and thus mimic the phase-advancing effects of light observed in *V1a*^*-/-*^*; V1b*^*-/-*^ mice. How re-entrainment to delays is enhanced in *V1a*^*-/-*^*; V1b*^*-/-*^ mice is still not known but it appears to be independent of Tenm3.

VIP-AVP connectivity plays a critical role in regulating the sensitivity of the SCN to light. Under normal circumstances, the master circadian clock processes light information and responds with gradual changes, coordinating numerous tissues and hormones to work in synchrony [6]. It would be evolutionarily problematic for the SCN pacemaker to be hypersensitive to subtle changes in light signaling. Here, we demonstrate that Tenm3 is a necessary component of VIP-AVP signaling, maintaining SCN sensitivity to light which is critical for physiological interactions with the environmental light/dark cycle. Our data support the notion that Tenm3 is not exclusive to image-forming visual system circuit assembly and function, and we show here for the first time it also plays a key role in establishing neuronal connectivity in a non-image-forming visual brain region.

## Materials and methods

### Ethics statement

All animals were handled in accordance with guidelines from the Animal Care and Use Committees of Johns Hopkins University School of Medicine (Protocol number MO23M68) and the National Institute of Mental Health (Protocol number SLCR-01). All efforts were made to minimize animal pain and the number of animals used.

### Animals

The following mice were described previously: *Opn4*^*Cre/+*^ [32] and *Brn3b*^*zDTA/+*^ [22]. *Opn4*^*lacZ/+*^ mice [33] were a gift from Dr. King-Wai Yau (Johns Hopkins University). *Tenm3*^*+/-*^ [18] and *Tenm3*^*fl/+*^ [14] mice were gifts from Dr. Liqun Luo (Stanford University).

The following mice were obtained from the Jackson Laboratory: *Rosa26*^*fsTRAP/fsTRAP*^ (stock number 022367), *ROSA*^*Synaptophysin-tdTomato/+*^ (stock number 012570), *VIP*^*Cre/Cre*^ (stock number 010908), and *C57Bl/6J* (stock number 000664).

The day of birth was designated as P0. Mice of both sexes were used in all experiments and were housed in a 12:12 light-dark (LD) cycle except where indicated.

### RNAscope *in situ* hybridization

Fluorescent *in situ* hybridization was performed on P5 *C57Bl/6J* retina or brain sections. Mice were transcardially perfused with 1× phosphate-buffered saline (PBS) followed by 4% paraformaldehyde (PFA) in 1× PBS. All tissue was fixed in 4% PFA overnight at 4°C and then washed the following day several times with 1× PBS. A hole was made in the cornea of fixed eyes prior to cryopreservation overnight at 4°C in 1× PBS containing 30% sucrose (w/v). Fixed brains were also cryopreserved overnight at 4°C in 1× PBS containing 30% sucrose (w/v). Cryopreserved tissue was embedded in Neg-50 frozen section medium (Richard-Allan Scientific, 6502) and sectioned using a cryostat at a thickness of 14 μm.

*In situ* hybridization was performed using the RNAscope Fluorescent Multiplex Reagent Kit v1 (Advanced Cell Diagnostics) according to the manufacturer’s instructions. The following probes were used in this study: *Opn4-C2*, *Pou4f2-C3*, *Tenm3-C1*, *AVP-C2*, and *VIP-C3*.

### Immunohistochemistry (brain)

Mice were transcardially perfused with 1× PBS followed by 4% PFA in 1× PBS. Dissected brains were fixed in 4% PFA for 4 h (P40) or overnight (>P56) at 4°C. All fixed tissue was washed several times with 1× PBS. Brains were then embedded in 3% (w/v) agarose and a vibratome was used to collect coronal sections at a thickness of 100 μm.

Brain sections were blocked for 1 h at room temperature in 1× PBS, 0.5% Triton X-100, and 10% goat serum. Tissue was then incubated in primary antibody diluted in 1× PBS, 0.5% Triton X-100, and 10% goat serum for 3 days at 4°C and washed the next day for several hours with 1× PBS, 0.5% TritonX-100 at room temperature. Tissue was incubated in secondary antibody diluted in 1× PBS, 0.5% Triton X-100, and 10% goat serum overnight at 4°C and washed for several hours the next day with 1× PBS, 0.5% TritonX-100 at room temperature. Brain sections were then mounted and imaged with a Zeiss LSM 700 confocal microscope.

Primary antibodies used include: chicken anti-β-Galactosidase (1:500, Aves, BGL-1040), chicken Y-RAN RFP [34] (1:500, produced in Kolodkin laboratory by Dr. Randal Hand), rabbit anti-AVP (1:500, Immunostar, 20069).

### Light-responsive immunohistochemistry (pH3)

*Tenm3*^*+/-*^ and *Tenm3*^*-/-*^ mice were entrained for a minimum of 1 week to a 12:12 LD schedule under a light intensity of either 100 or 10 lux. Mice were then released into dark-dark (DD) for 1 day prior to light pulse. Two hours before the end of their active phase, circadian time 22h (CT22), mice received a 15-min light pulse at their respective light intensities. Mice were anesthetized and perfused 15 min after the end of the light pulse treatment. To limit light exposure after light pulse, the heads of anesthetized mice were covered with a light tight hood until perfusions were completed. Additionally, perfusions occurred in a dark room using dim red light. Brains were removed and postfixed in 4% PFA overnight at 4°C. Brains were subsequently cryoprotected in a 30% sucrose solution in 1× PBS for approximately 48 h then mounted and frozen in OCT. Brains were sectioned at a 40 μm thickness on a cryostat and SCN sections were collected (approximately 4 to 6 sections from rostral to caudal).

To visualize light-dependent cell activation in the SCN, sections were subjected to immunofluorescence staining to label cells undergoing phosphorylation of histone 3 (pH3) as described previously [23]. Briefly, sections were first washed in 0.5% PBST 3 times. SCN sections then underwent antigen retrieval in sodium citrate buffer (10 mM sodium citrate, 0.05% tween 20 (pH 6.0)) for 30 min at 80°C. Once cooled to room temperature, sections were washed then blocked in 10% bovine serum albumin (BSA in 0.5% PBST) for 1 h. Sections were incubated in primary antibody Phospho-Histone H3 rabbit mAb (1:1,000, Cell Signaling Technology, Danvers, Massachusetts, United States of America) diluted in 2.5% BSA (0.5% PBST) overnight at 4°C. Sections were then washed 3 times in 0.5% PBST then incubated in secondary antibody donkey anti-rabbit Alexa Fluor 488 (1:500, Thermo Fisher Scientific, 1:500) for 1 h. Finally, sections were washed in 0.5% PBST, counterstained with DAPI (Thermo Fisher Scientific) and mounted on slides using Fluoromount-G (Thermo Fisher Scientific).

### pH3+ cell quantification

As described in a previous publication [23], the SCN was identified by dense nuclear (DAPI) staining in maximum projections of confocal z-stacked images. Tight boundaries were drawn around each SCN hemisphere to approximate area and facilitate cell counting. For each hemisphere, the area, perimeter, and relative shape of the SCN were used to match rostral to caudal sections across mice. pH3-positive cells within the SCN boundary in middle (core containing) SCN sections were then manually counted with the multipoint tool in ImageJ (FIJI) and normalized by area established by SCN boundaries or by a static circular area that approximated the core only. Quantification is reported as the average pH3+ cell density from core containing sections per mouse.

### SCN innervation analysis

For experiments with *Opn4*^*lacZ/+*^ mice, SCN axon innervation was determined by calculating the fluorescence intensity of β-Galactosidase. Briefly, each hemisphere of the SCN was cropped to a size of 290 × 430 μm. This region was further subdivided into 4 equal quadrants (dorso-lateral, dorso-medial, ventro-lateral, and ventro-medial) and the integrated density of β-Galactosidase was determined for each region. To determine the total ventro-lateral or ventro-medial SCN innervation in each animal, the integrated density values of 4 consecutive sections were summed. No alterations to image brightness or contrast were made prior to quantification. Control integrated density values can be highly variable between staining cohorts. Therefore, for experiments involving multiple cohorts of animals, values were normalized to controls within each group and the normalized value was reported.

### Stereotaxic injections

Mice were anesthetized with isoflurane and given systemic analgesics (meloxicam 0.1 mg/kg; Boehringer Ingelheim). A heating pad was used to maintain mouse body temperature during the surgery. Injections were performed using a microinjector (Nanojector III, Drummond Scientific Company) and an Angle-Two small animal stereotaxic instrument (Leica). Specifically, a microcapillary pipette was pulled and loaded with a 1:20 mixture of *AAVretro-hSyn-eGFP* (Addgene, #50465-AAVrg) and *AAV8-hSyn-mCherry* (Addgene, #114472-AAV8) which was stereotaxically delivered to the SCN in both hemispheres. Coordinates were determined in *Tenm3*^*+/-*^ mice to be as follows: ML: +/− 0.2, AP: 0.15, DV: −5.45, Tilt: +/− 4.2°. A local analgesic was administered at the site of incision (lidocaine, 2 mg/kg; Fresenius Kabi). For anatomical analysis, mice were transcardially perfused with 4% paraformaldehyde 3 weeks after injection. Brains were dissected and postfixed in 4% PFA overnight before being cryoprotected in a 30% sucrose solution for 2 days. Once brains reached equilibrium in the sucrose solution, they were sectioned on a cryostat, briefly stained with DAPI, and examined under a confocal microscope to determine injection success. Mice without correct targeting of viral vectors were excluded from this study. After perfusion, retinas were dissected and buffered in a 6% sucrose solution before being postfixed in 4% PFA for 1 h and subsequently transferred to PBS for processing.

### Immunohistochemistry (retina)

Wholemount retinas were blocked overnight in 3% goat serum (1% PBS Triton X-100) at 4°C. Retinas were then incubated in primary antibody (1:500 Rabbit anti-Melanopsin; Advanced Targeting Systems, AB-N38, lot# 140–85, and 1:1,000 Chicken anti-GFP; Abcam, ab13970) diluted in 1% goat serum, 1% PBST for 3 days at 4°C. Retinas were then washed in 1% PBST overnight and incubated in secondary antibodies goat anti-chicken Alexa Fluor 488 and goat anti-rabbit Alexa Fluor 555 (1:1,000, Thermo Fisher) the following day for 24 h. Retinas were washed in 1% PBST overnight before being mounted on superfrost plus microscope slides and imaged.

### Quantification of retrogradely labeled RGCs

Wholemount retinas were imaged on a Nikon A1 inverted confocal microscope. Tiled scans of the whole retina were recorded at 10× magnification before systematically imaging retrogradely labeled eGFP-positive cells 1 laser channel at a time under a 25× oil objective. Z-stack images with a 1 micron step size were recorded for eGFP-positive cells. Cells were determined to be melanopsin-positive if the melanopsin signal overlapped or tightly bound the eGFP-positive cell and could be distinguished from background signal throughout the z-stack. To account for variation of viral injection position, cell counts were normalized to total cells counted per retina and percentage of GFP-, melanopsin-double positive and GFP-positive only out of total number of retrogradely label cells was reported.

### Wheel-running activity

Mice were single housed in cages, each containing a 4.5-inch running wheel. Wheel-running activity was monitored with VitalView software (MiniMitter) and analyses of wheel-running activity were calculated using ClockLab software (Actimetrics, version 6.0.53).

Mice were initially placed under a 12:12 LD photoentrainment paradigm at 1,000 Lux until they entrained to the 12:12 light schedule (at least 10 days). Once entrained, light onset was advanced by 6 h and mice were allowed to re-entrain to the new schedule for 10 days. Light onset was subsequently delayed by 6 h and mice were allowed to re-entrain to the new schedule. At the end of the phase shifting experiment, mice were released to constant darkness for at least 14 days to measure the endogenous circadian period.

Time to re-entrain to phase advances was measured from the beginning of phase advance to the first day where activity onset (usually coinciding with the beginning of the dark phase) was consistent with the time of activity onset for consecutive days. Similarly, time to re-entrain to phase delays was measured as the time required for activity offset (or onset) to occur at the same time for consecutive days after a phase delay.

### Single-cell RNA sequencing

*Opn4*^*Cre/+*^*; Brn3b*^*zDta/+*^*; Rosa26*^*fsTRAP/+*^ mice were sacrificed at P1 or P5 and retinas were rapidly dissected in cold Hibernate A medium lacking Ca^2+^, Mg^2+^, and phenol red (BrainBits). Retinas were then dissociated in papain (Worthington Biochemical, LS003126), 5.5 mM Cysteine-HCl, and 1.1 mM EDTA dissolved in Hibernate A medium (lacking Ca^2+^, Mg^2+^, and phenol red) for 7 min at 37°C. Cell suspension was passed through a 40 μm cell strainer (Falcon, 352340) and incubated for 30 min on ice with Brilliant Violet (BV)-421 anti-mouse CD90.2 antibody (BioLegend, 105341, 1:300), which binds to RGCs and provides an additional selection marker during FACS. The cells were then centrifuged and resuspended in 0.4% BSA dissolved in Hibernate A medium (lacking Ca^2+^, Mg^2+^, and phenol red). Cells were again centrifuged and resuspended in 2,000 U/ml DNase I (New England Biolabs, M0303S) in Hibernate A medium (lacking Ca^2+^, Mg^2+^, and phenol red) prior to cell sorting.

Individual GFP^+^, BV-421^+^ cells were sorted into a lysis solution consisting of 3,500 U/ml RNase inhibitor (New England Biolabs, M0314S), 140 U/ml DNase I, and 0.17% v/v Triton X-100 in water using a MoFlo Legacy cell sorter (Beckman Coulter). The Smart-seq2 protocol [35] was used to prepare single-cell DNA libraries for sequencing on an Illumina NextSeq 500 sequencer (75 bp paired-end reads, 400 million total reads).

FASTQ files were aligned to the GRCm38.p6 (mm10) mouse reference genome using HISAT2 version 2.1.0 [36]. The resulting SAM files were converted to BAM files using SAMtools version 1.9 [37]. Transcript abundance was estimated using Cuffquant and normalization was performed using Cuffnorm in Cufflinks version 2.2.1 [38]. All analyses were performed using Seurat [39] in R version 3.6.2 (The R foundation for Statistical Computing).

### Statistical analysis

All graphs except for those displaying scRNA-seq results were generated using Prism version 9 (GraphPad). Graphs showing scRNA-seq results were generated using the ggplot2 package in R version 3.6.2 (The R foundation for Statistical Computing). Student’s *t* tests and analyses of variance (ANOVAs) were performed in Prism. The threshold for statistical significance was defined as *p* < 0.05.

## Supporting information

S1 Fig *Tenm3* is very weakly expressed by *Pou4f2*-negative ipRGCs. (A) *Tenm3* expression is very weak in P5 *Pou4f2*-negative ipRGCs (dotted white circles, arrows) and higher in *Pou4f2*-positive ipRGCs (dotted white circles, no arrows). *Opn4*-positive cells expressing the lowest to highest *Pou4f2* levels are indicated by groups 1–4. Lines represent mean. Symbols represent individual cells (see S1 Data). *n* = 3 mice and 21–25 *Opn4*-positive cells/mouse. Statistics: unpaired *t* test. ****p < 0.0001. Scale bar = 25 μm. (B) Single GFP-positive cells were collected from P1 and P5 *Opn4*^*Cre/+*^*; Brn3b*^*zDta/+*^*; Rosa26*^*fsTRAP/+*^ retinas, time points before and during ipRGC innervation of the SCN, respectively. (C) UMAP unsupervised clustering reveals 3 populations of cells. (D) Feature plots of known marker genes *Rbpms*, *Eomes*, *Opn4*, and *Pou4f2*. Cluster 2 contains the *Opn4*-positive, *Pou4f2*-negative ipRGCs that innervate the SCN. (E) scRNA-seq reveals *Tenm3* is not expressed by *Pou4f2*-negative ipRGCs (blue dotted oval) and is only highly expressed in very few other ipRGCs from this dataset.
(TIF)

S2 Fig Innervation of the IGL and OPN are normal in *Tenm3*^*-/-*^*; Opn4*^*lacZ/+*^ mice. (A) β-Galactosidase labeling in the IGL and OPN of *Tenm3*^*+/-*^*; Opn4*^*lacZ/+*^ and *Tenm3*^*-/-*^*; Opn4*^*lacZ/+*^ mice at P40. Scale bar = 100 μm. (B, C) Innervation of the IGL and OPN in *Tenm3*^*-/-*^*; Opn4*^*lacZ/+*^ mice is similar to *Tenm3*^*+/-*^*; Opn4*^*lacZ/+*^ animals. Lines represent mean and SD. Symbols represent individual mice (see S1 Data). Statistics: unpaired *t* test.
(TIF)

S3 Fig Tenm3 is not required in ipRGCs for proper axon innervation of the SCN. (A) β-Galactosidase labeling in the SCN of *Opn4*^*Cre/+*^*; Tenm3*^*fl/-*^ conditional mutants is similar to control animals at P40. (B, C) Both the ventro-medial and ventro-lateral SCN are normally innervated in *Opn4*^*Cre/+*^*; Tenm3*^*fl/-*^ conditional mutants. Lines represent mean and SD. Symbols represent individual mice (see S1 Data). Statistics: unpaired *t* test. Scale bar = 100 μm.
(TIF)

S4 Fig RGCs do not increase innervation to the SCN in the absence of Tenm3 and ipRGC innervation. (A) Schematic of the viral tracing strategy to retrogradely label RGCs in *Tenm3*^*-/-*^ mice. Viral expression was confirmed for both mCherry and retro-eGFP in brain sections of *Tenm3*^*+/-*^ (B, D) and *Tenm3*^*-/-*^ (E) mice. Scale bar 100 μm. Wholemount retinas of *Tenm3*^+/-^ (C, D) and *Tenm3*^-/-^ mice (F) contained RGCs that were immunopositive for eGFP and melanopsin (orange arrows) or eGFP only (white arrows). Black scale bar = 1,000 μm. White scale bar = 100 μm. nsb = nonspecific binding. (G) The percentage of melanopsin-positive and negative retrogradely labeled RGCs was consistent across mice. In the *Tenm3*^*-/-*^ mouse, 72.4% of cells were GFP-positive and melanopsin-positive (*n* = 21). Similarly, double positive cells accounted for 78.7% (*n* = 37) and 75% (*n* = 39) of total retrogradely labeled cells in *Tenm3*^*+/-*^ mice (see S1 Data).
(TIF)

S5 Fig pH3 induction is primarily in the core and is light dependent. (A) Representative maximum projection images of the phosphorylation of histone H3 after a 15-min light pulse at 10 lux for *Tenm3*^*+/-*^ and *Tenm3*^*-/-*^ mice. (B) Quantification of the light-induced phosphorylation indicates that a significant majority of the phosphorylation occurs in the core (*p* = 0.0072) and in the shell (*p* = 0.0278) (see S1 Data). (C) Maximum projection images of mice that did not receive a 15-min light pulse prior to perfusion show little to no phosphorylation of H3, indicating that this phosphorylation event is primarily light dependent in the SCN and is not present due to the loss of Tenm3.
(TIF)

S6 Fig Additional actograms of circadian photoentrainment at 1,000 lux. (A) Individual double-plotted wheel-running activity under 1,000 lux for *Tenm3*^*+/-*^ and (B) *Opn4*^*-/-*^ mice which normally re-entrain to 6-h phase advances and delays within 4–6 days under 12:12 LD conditions. Global loss of Tenm3 in (C) *Tenm3*^*-/-*^ and (D) *Tenm3*^*-/-*^*; Opn4*^*-/-*^ mice robustly show an accelerated re-entrainment after a 6-h phase advance, but not after phase delays. (E) Quantification of the time to re-entrain to phase advances at 1,000 lux across genotypes shows a significant difference between *Tenm3*^*-/-*^ and *Tenm3*^*+/-*^ mice (*p* = 0.0206) or *Tenm3*^*-/-*^ and *Opn4*^*-/-*^ mice (*p* = 0.0016) as well as between *Opn4*^*-/-*^ and *Tenm3*^*-/-*^*; Opn4*^*-/-*^ mice (*p* = 0.0347). There was no difference found in time to re-entrain to phase advances between *Tenm3*^*+/-*^ mice and *Opn4*^*-/-*^ mice. (F, G) No significant difference was found for time to re-entrain to phase delays as measured from the onset or offset of activity. (H) Additionally, no significant difference was found in wheel-running period length under DD. Error represents standard deviation (see S2 Data). One-way ANOVA with Tukey’s correction for multiple comparisons. Arrows denote observed day of re-entrainment to advances and delays (measured by onset and offset of activity). Red lines represent loss of data.
(TIF)

S7 Fig Additional actograms of circadian photoentrainment at 10 lux. (A) At 10 lux, *Tenm3*^*+/-*^ and *Tenm3*^*-/-*^ (B) mice take longer to re-entrain to phase changes. Arrows denote observed day of re-entrainment to advances and delays (measured by onset and offset of activity). See S3 Data for values.
(TIF)

S1 Data Individual quantitative observations underlying data summarized in Figs 1–3 and S1–S5. (XLSX)

S2 Data 1,000 lux wheel-running count data underlying Figs 4 and S6. (XLSX)

S3 Data 10 lux wheel-running count data underlying Figs 4 and S7. (XLSX)

## Abbreviations

AVP: arginine vasopressin
BSA: bovine serum albumin
CNS: central nervous system
CT: circadian time
DD: dark-dark
DKO: double knockout
GRP: gastrin releasing-peptide
IGL: intergeniculate leaflet
ipRGC: intrinsically photosensitive retinal ganglion cell
LD: light-dark
OPN: olivary pretectal nucleus
PBS: phosphate buffered saline
RGC: retinal ganglion cell
SC: superior colliculus
SCN: suprachiasmatic nucleus
scRNA-seq: single-cell RNA sequencing
VIP: vasoactive intestinal peptide
