# Supplementary figures and images for: Teneurin-3 regulates the generation of non-image-forming visual circuitry and responsiveness to light in the suprachiasmatic nucleus
Source: PLoS Biol. 2023 Dec 4;21(12):e3002412. doi: 10.1371/journal.pbio.3002412 (PMC10729976; doi:10.1371/journal.pbio.3002412)

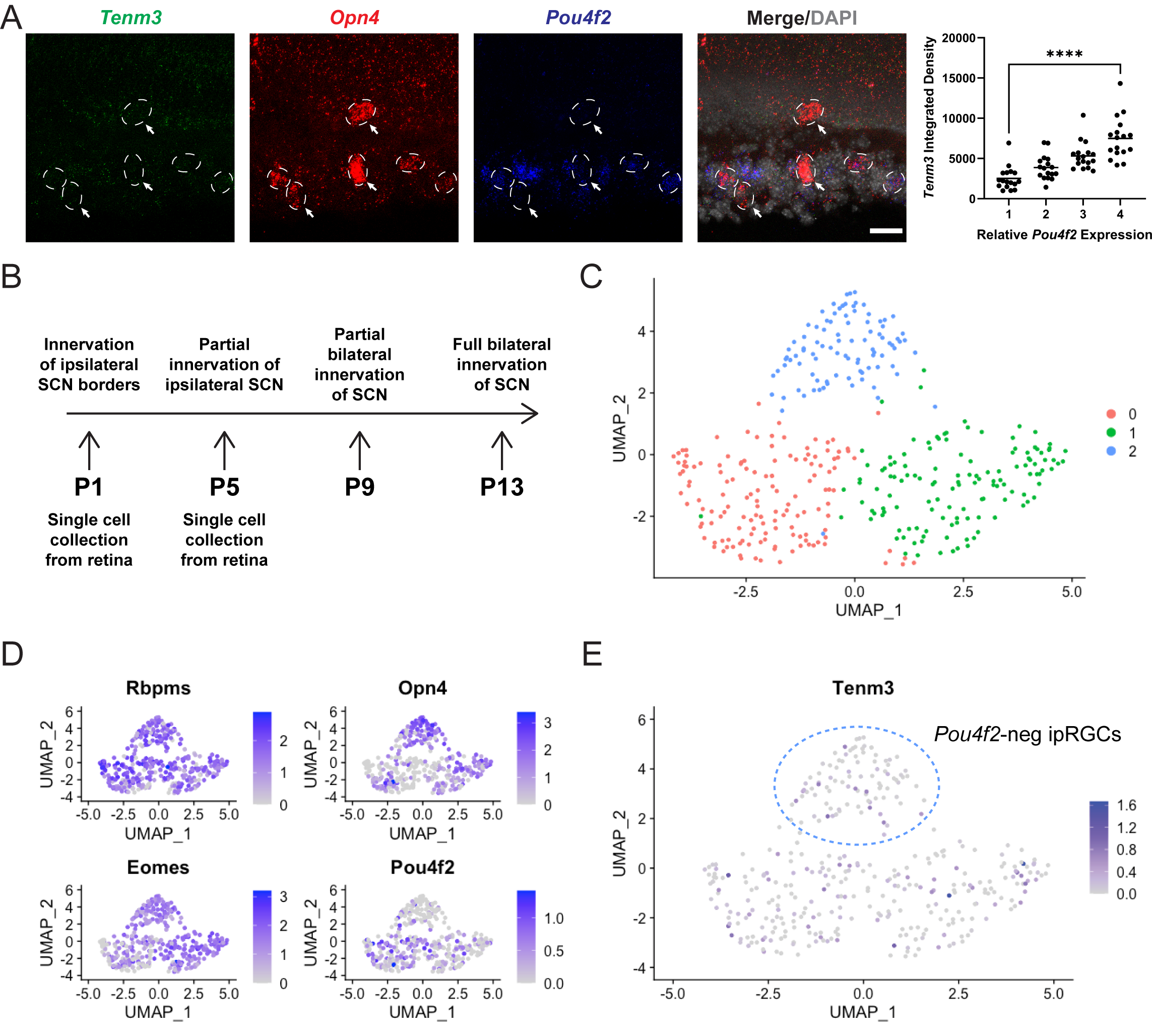

Supplement: S1 Fig — (A) Tenm3 expression is very weak in P5 Pou4f2-negative ipRGCs (dotted white circles, arrows) and higher in Pou4f2-positive ipRGCs (dotted white circles, no arrows). Opn4-positive cells expressing the lowest to highest Pou4f2 levels are indicated by groups 1–4. Lines represent mean. Symbols represent individual cells (see S1 Data). n = 3 mice and 21–25 Opn4-positive cells/mouse. Statistics: unpaired t test. ****p < 0.0001. Scale bar = 25 μm. (B) Single GFP-positive cells were collected from P1 and P5 Opn4Cre/+; Brn3bzDta/+; Rosa26fsTRAP/+ retinas, time points before and during ipRGC innervation of the SCN, respectively. (C) UMAP unsupervised clustering reveals 3 populations of cells. (D) Feature plots of known marker genes Rbpms, Eomes, Opn4, and Pou4f2. Cluster 2 contains the Opn4-positive, Pou4f2-negative ipRGCs that innervate the SCN. (E) scRNA-seq reveals Tenm3 is not expressed by Pou4f2-negative ipRGCs (blue dotted oval) and is only highly expressed in very few other ipRGCs from this dataset. (TIF) [file pbio.3002412.s001.tif]

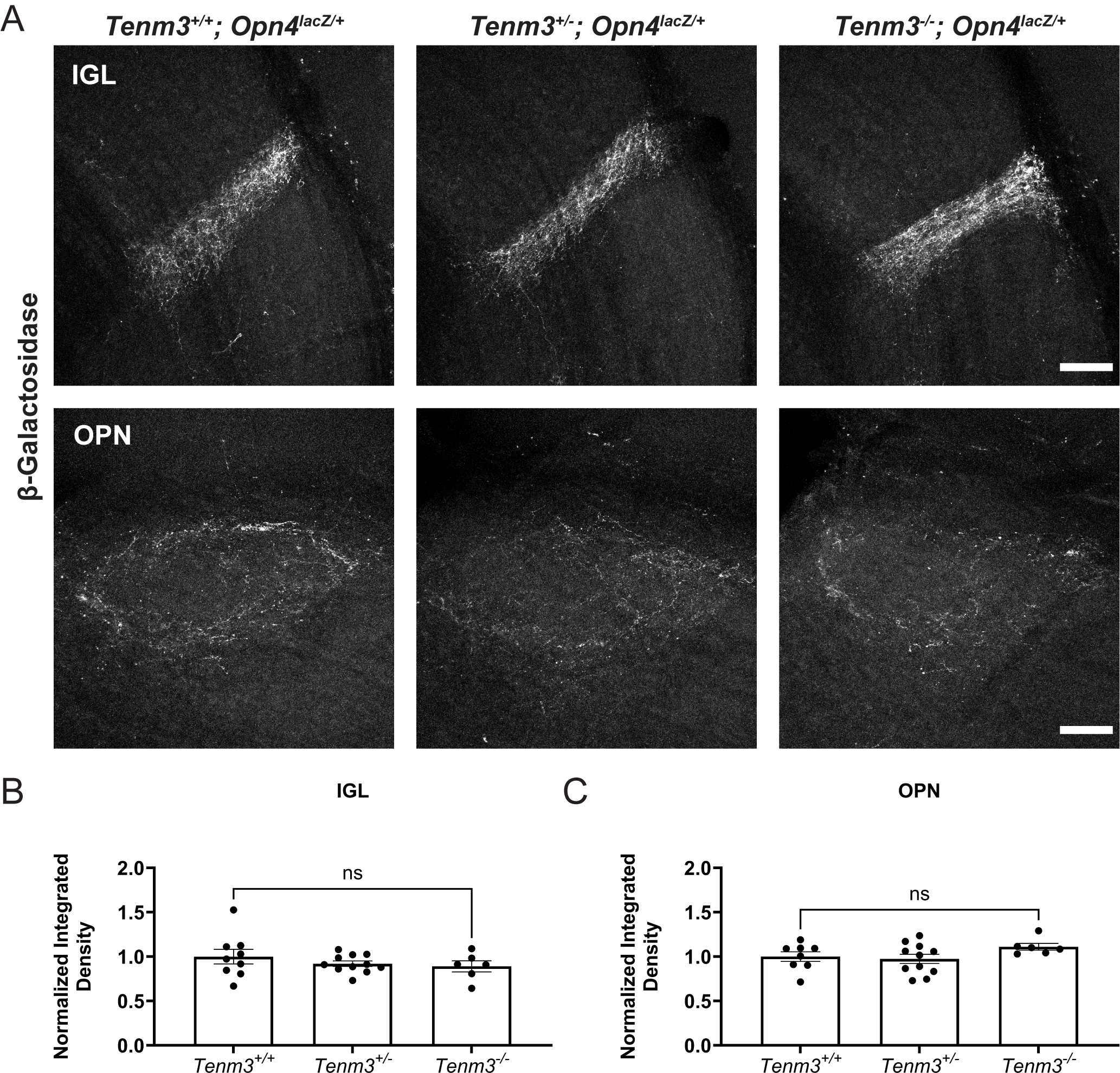

Supplement: S2 Fig — (A) β-Galactosidase labeling in the IGL and OPN of Tenm3+/-; Opn4lacZ/+ and Tenm3-/-; Opn4lacZ/+ mice at P40. Scale bar = 100 μm. (B, C) Innervation of the IGL and OPN in Tenm3-/-; Opn4lacZ/+ mice is similar to Tenm3+/-; Opn4lacZ/+ animals. Lines represent mean and SD. Symbols represent individual mice (see S1 Data). Statistics: unpaired t test. (TIF) [file pbio.3002412.s002.tif]

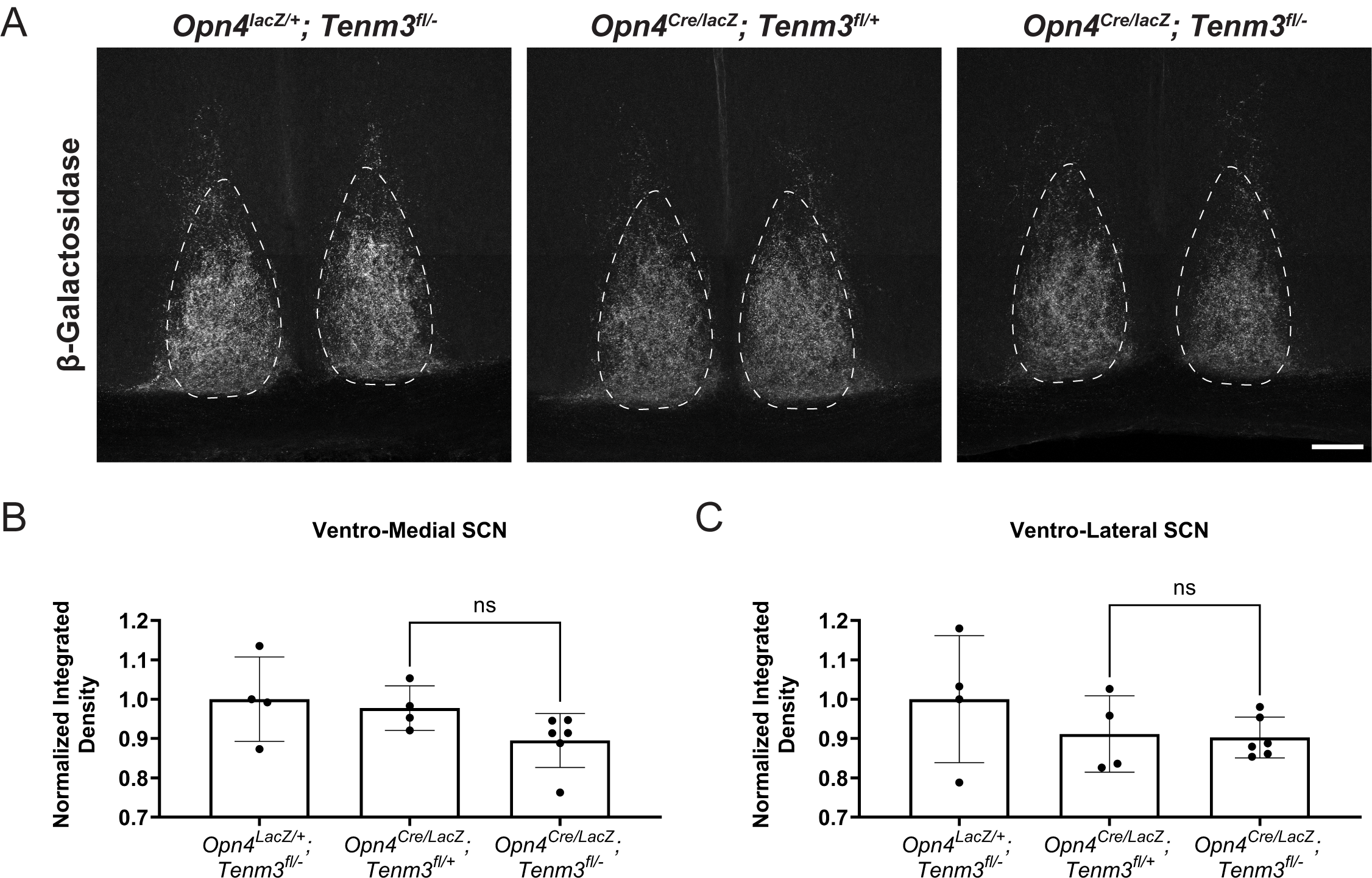

Supplement: S3 Fig — (A) β-Galactosidase labeling in the SCN of Opn4Cre/+; Tenm3fl/- conditional mutants is similar to control animals at P40. (B, C) Both the ventro-medial and ventro-lateral SCN are normally innervated in Opn4Cre/+; Tenm3fl/- conditional mutants. Lines represent mean and SD. Symbols represent individual mice (see S1 Data). Statistics: unpaired t test. Scale bar = 100 μm. (TIF) [file pbio.3002412.s003.tif]

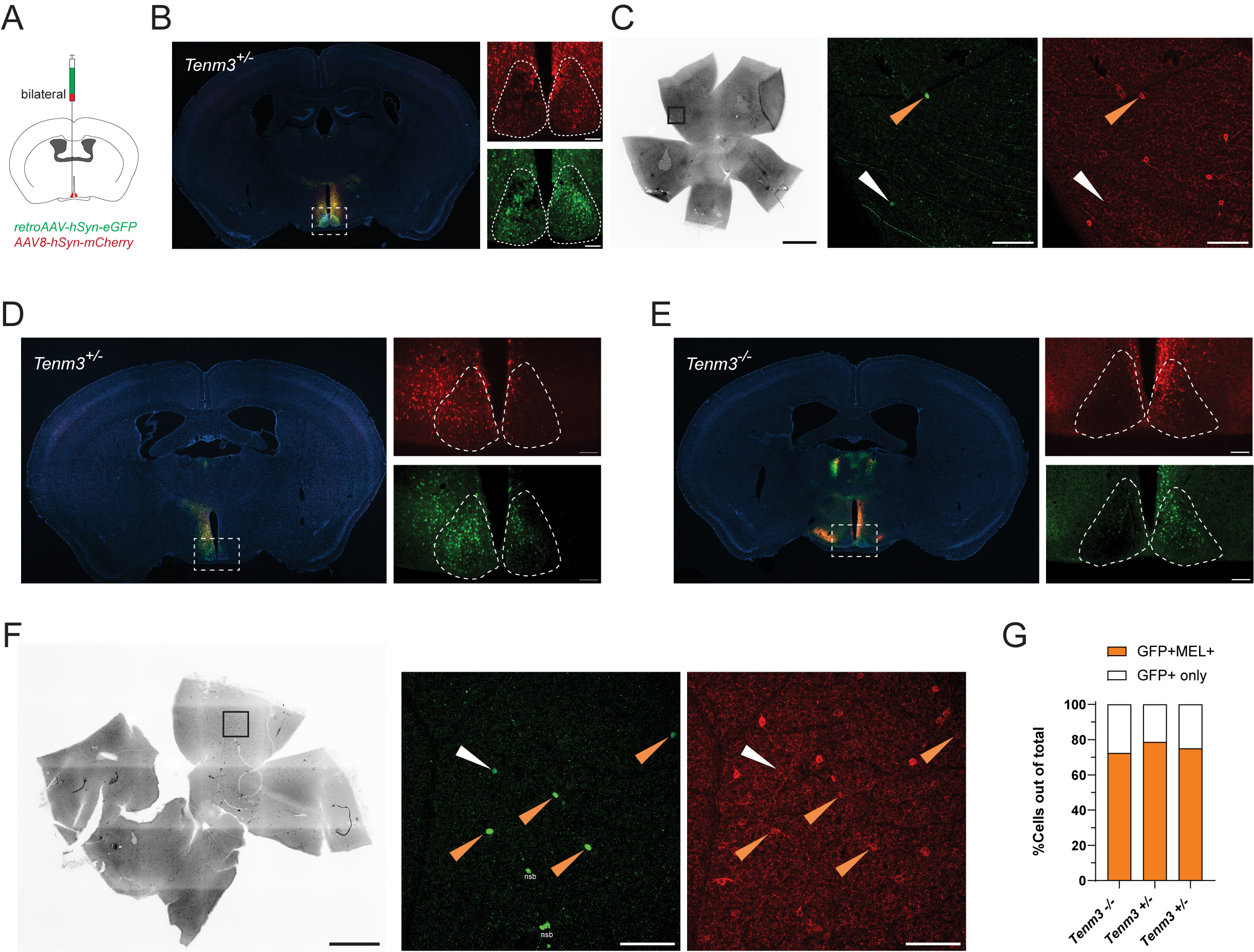

Supplement: S4 Fig — (A) Schematic of the viral tracing strategy to retrogradely label RGCs in Tenm3-/- mice. Viral expression was confirmed for both mCherry and retro-eGFP in brain sections of Tenm3+/- (B, D) and Tenm3-/- (E) mice. Scale bar 100 μm. Wholemount retinas of Tenm3+/- (C, D) and Tenm3-/- mice (F) contained RGCs that were immunopositive for eGFP and melanopsin (orange arrows) or eGFP only (white arrows). Black scale bar = 1,000 μm. White scale bar = 100 μm. nsb = nonspecific binding. (G) The percentage of melanopsin-positive and negative retrogradely labeled RGCs was consistent across mice. In the Tenm3-/- mouse, 72.4% of cells were GFP-positive and melanopsin-positive (n = 21). Similarly, double positive cells accounted for 78.7% (n = 37) and 75% (n = 39) of total retrogradely labeled cells in Tenm3+/- mice (see S1 Data). (TIF) [file pbio.3002412.s004.tif]

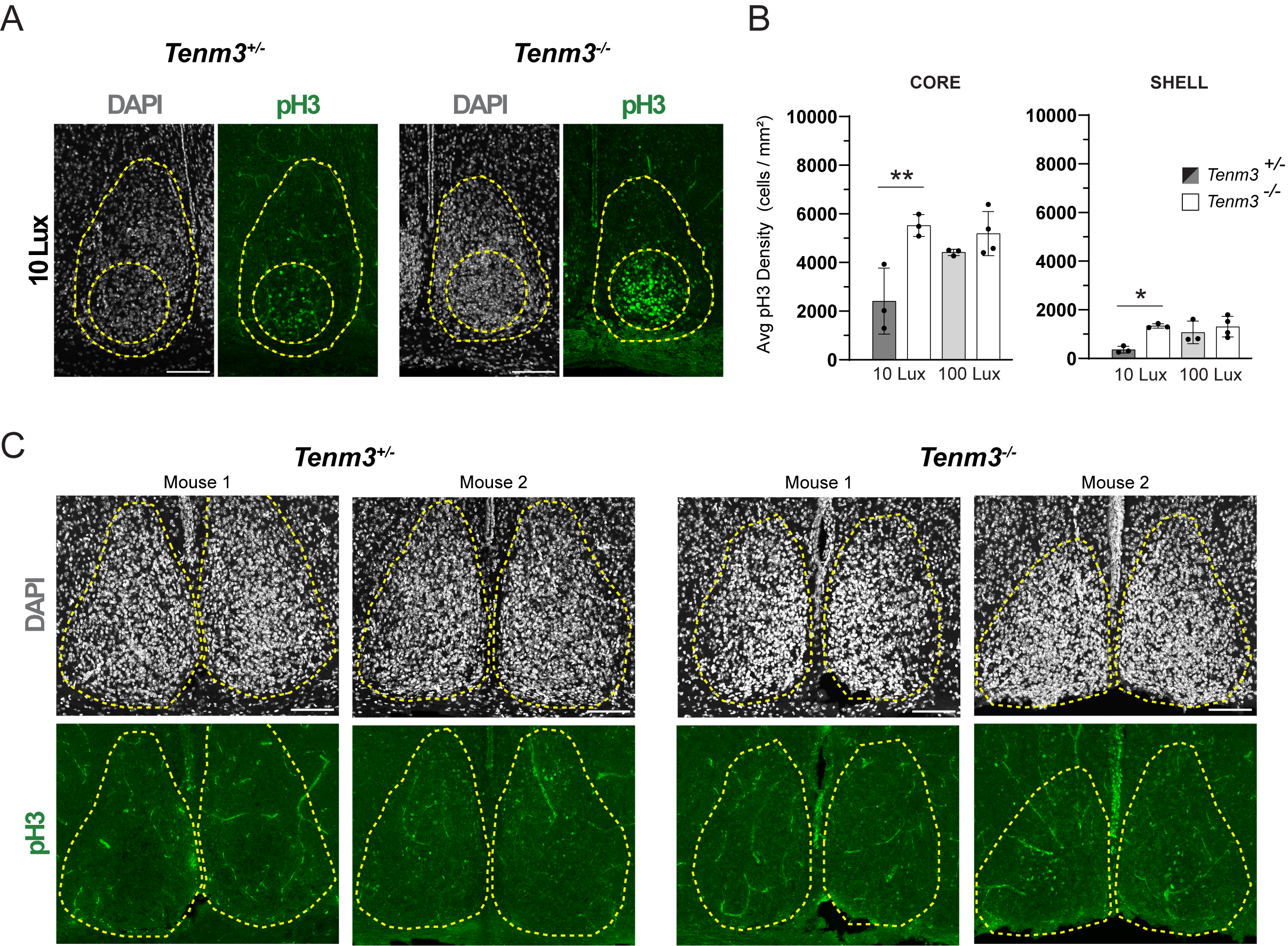

Supplement: S5 Fig — (A) Representative maximum projection images of the phosphorylation of histone H3 after a 15-min light pulse at 10 lux for Tenm3+/- and Tenm3-/- mice. (B) Quantification of the light-induced phosphorylation indicates that a significant majority of the phosphorylation occurs in the core (p = 0.0072) and in the shell (p = 0.0278) (see S1 Data). (C) Maximum projection images of mice that did not receive a 15-min light pulse prior to perfusion show little to no phosphorylation of H3, indicating that this phosphorylation event is primarily light dependent in the SCN and is not present due to the loss of Tenm3. (TIF) [file pbio.3002412.s005.tif]

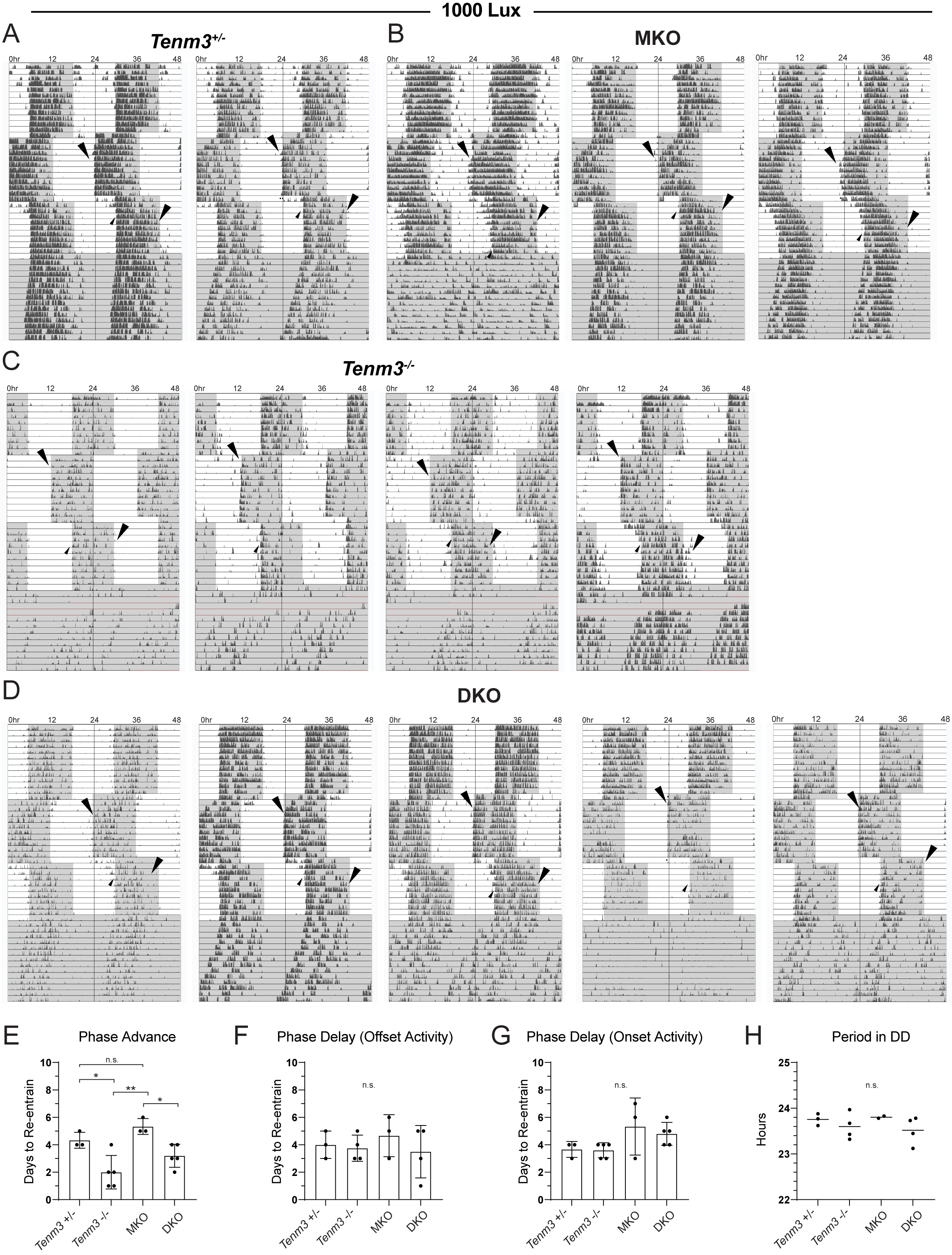

Supplement: S6 Fig — (A) Individual double-plotted wheel-running activity under 1,000 lux for Tenm3+/- and (B) Opn4-/- mice which normally re-entrain to 6-h phase advances and delays within 4–6 days under 12:12 LD conditions. Global loss of Tenm3 in (C) Tenm3-/- and (D) Tenm3-/-; Opn4-/- mice robustly show an accelerated re-entrainment after a 6-h phase advance, but not after phase delays. (E) Quantification of the time to re-entrain to phase advances at 1,000 lux across genotypes shows a significant difference between Tenm3-/- and Tenm3+/- mice (p = 0.0206) or Tenm3-/- and Opn4-/- mice (p = 0.0016) as well as between Opn4-/- and Tenm3-/-; Opn4-/- mice (p = 0.0347). There was no difference found in time to re-entrain to phase advances between Tenm3+/- mice and Opn4-/- mice. (F, G) No significant difference was found for time to re-entrain to phase delays as measured from the onset or offset of activity. (H) Additionally, no significant difference was found in wheel-running period length under DD. Error represents standard deviation (see S2 Data). One-way ANOVA with Tukey’s correction for multiple comparisons. Arrows denote observed day of re-entrainment to advances and delays (measured by onset and offset of activity). Red lines represent loss of data. (TIF) [file pbio.3002412.s006.tif]

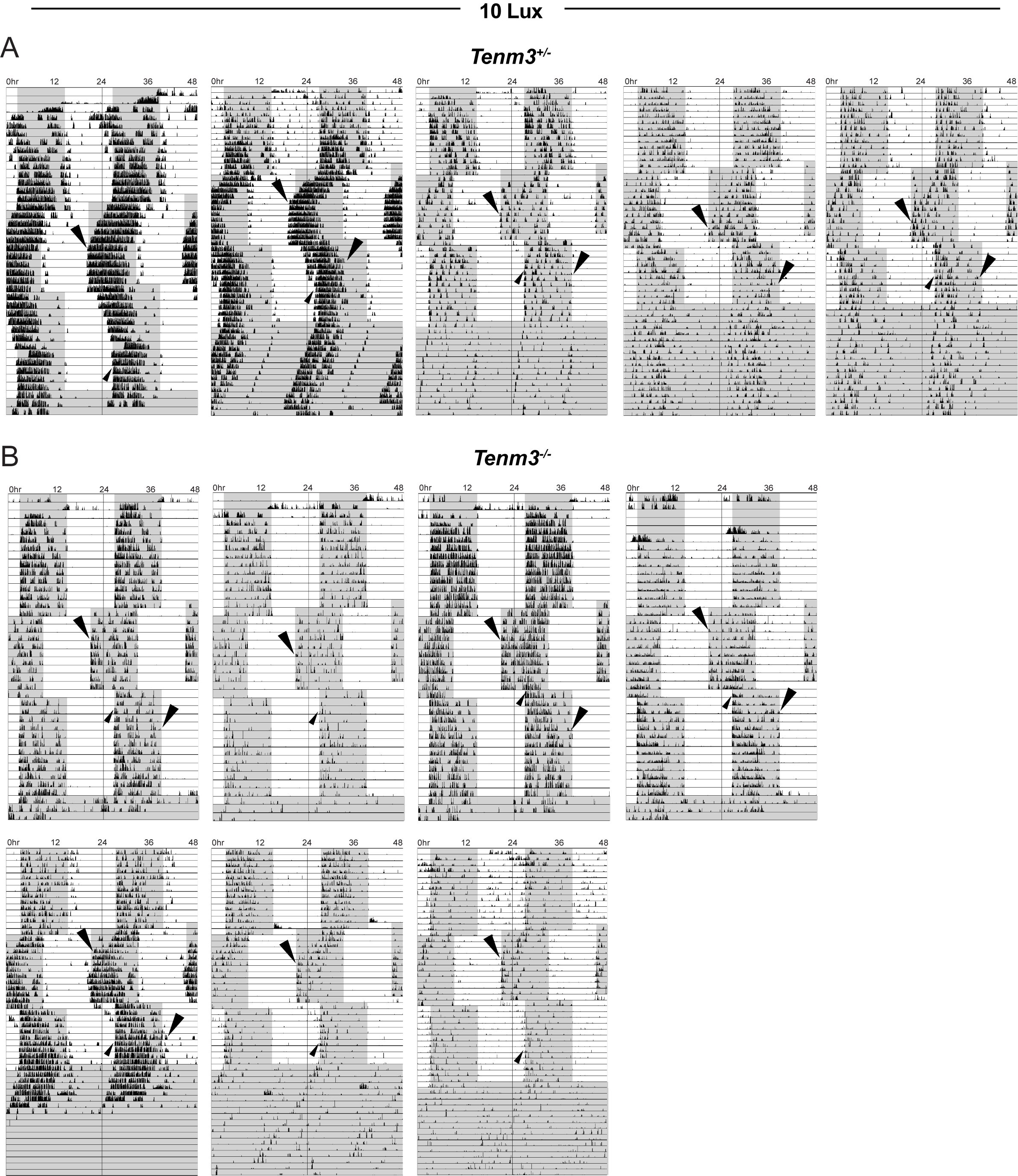

Supplement: S7 Fig — (A) At 10 lux, Tenm3+/- and Tenm3-/- (B) mice take longer to re-entrain to phase changes. Arrows denote observed day of re-entrainment to advances and delays (measured by onset and offset of activity). See S3 Data for values. (TIF) [file pbio.3002412.s007.tif]
